# Cases of Prolonged Lactation

**Published:** 1848-11

**Authors:** 


*Cases of Prolonged Lactation*—Dr. Moir mentioned the case of a married lady, who, for certain reasons, was unable to nurse her own children, but in whom the secretion of milk was after each confinement unusually protracted, notwithstanding the means used to discuss it. Starvation, purgatives, diuretics, diaphoretics, and alteratives, as internal remedies, and local astringents of various kinds, were used, without in the least diminishing the secretion, or having any effect further than that of weakening the patient, so that latterly the only means employed were of a tonic nature, to sustain her strength. The secretion continued from one confinement till about the third month of the succeeding pregnancy, after which it almost ceased; after her second confinement it continued eighteen months ; after her third, twenty-four months ; after her fourth, twenty-five months , after the fifth, about twenty-four months, when she had a miscarriage, and since then has had no children. A circumstance that rendered this case peculiarly distressing was, that after her first confinement she suffered from a severe mammary abscess, which, by the practitioner then in attendance, was opened close to the nipple. From this wound the milk continually flowed, and as it never healed up, it was impossible to receive the milk from this breast into any convenient reservoir ; so that the lady was kept in a constant state of discomfort, her dress, notwithstanding the use of oiled silk, and M'Intosh cloth, being completely saturated with milk. The skin over the abdomen and left side was from the same cause much irritated, and in warm weather partially excoriated.

Dr. Peddie knew of a woman having nursed uninteruptedly for three years.—*Proceedings of Edinburgh Obstetric Society.*

